# Medication Beliefs and Adherence to Antiseizure Medications

**DOI:** 10.1155/2020/6718915

**Published:** 2020-10-23

**Authors:** Devender Bhalla, Elham Lotfalinezhad, Fatemeh Amini, Ahmad Delbari, Reza Fadaye-Vatan, Vida Saii, Kurosh Gharagozli

**Affiliations:** ^1^Iranian Epilepsy Association, Tehran, Iran; ^2^Sudan League of Epilepsy and Neurology (SLeN), Khartoum, Sudan; ^3^Nepal Interest Group of Epilepsy and Neurology (NiGEN), Kathmandu, Nepal; ^4^Pôle Universitaire Euclide Intergovernmental UN Treaty 49006/49007, Bangui, Central African Republic; ^5^Iranian Research Centre on Aging, University of Social Welfare and Rehabilitation Sciences, Tehran, Iran; ^6^Department of Health Education and Promotion, Tabriz University of Medical Sciences, Tabriz, Iran; ^7^Department of Neurology, Shahid Beheshti University of Medical Sciences, Tehran, Iran

## Abstract

**Introduction:**

The primary objective of our study was to determine the nature of medication beliefs and their association with adherence to antiseizure medications (ASMs) among elderly epilepsy patients. Our secondary objective was to enhance the psychometric properties and factor structure parameters of the Beliefs about Medications Questionnaire (BMQ) adapted to epilepsy and affected aged subjects.

**Methods:**

A population-based survey was performed in which older adults (≥60 years of age) were invited for a free face-to-face consultation with the specialists as well as for the collection of necessary data. The eligible subjects were those who are affected with epilepsy and having epileptic seizures of any type. In addition, the participants were required to be of any sex, currently under treatment with ASMs, resident of Tehran, and able and interested to participate independently. All were carefully examined with a reasonably detailed case-history examination. Two Persian questionnaires used were Medication Adherence Rating Scale (MARS) and BMQ. Those with a MARS score of ≥6 were considered as adherent to ASMs. All data were described in descriptive terms. We did a group comparison of means and proportions for all possible independent variables between adherents and nonadherents. Then, we did a hierarchical multiple linear regression. For this, independent variables were categorized into three different blocks: (a) sociodemographic block (Block-1), (b) treatment side-effect block (Block-2), and (c) BMQ block that included ten items of the BMQ scale (Block-3). We also did a forward step-wise linear regression by beginning with an empty model. We also estimated the psychometric properties and factor structure parameters of BMQ and its two subdomains.

**Results:**

Of all (*N* = 123, mean age: 63.3 years, 74.0% males), 78.0% were adherent (mean score: 7.0, 95% CI 6.2–7.8) to ASMs. The MARS scores were not different between males and females. The mean BMQ score was 23.4 (95% CI 19.8–27.0) with the mean need score of 20.0 (95% CI 18.0–22.0) and mean concern score of 16.5 (95% CI 14.3–18.7). A positive need-concern differential was 20.4%. Upon hierarchical regression, the adjusted *R*^2^ for Block-1 was 33.8%, and it was 53.8% for Block-2 and 92.2% for Block-3. Upon forward step-wise linear regression, we found that “ASMs disrupt my life” (*ß* −1.9, ES = −1.1, *p*=0.008) as the only belief associated with adherence. The alpha coefficient of BMQ was 0.81.

**Conclusions:**

Ours is one of the very few studies that evaluated medication beliefs and their association with adherence to ASMs among elderly epilepsy patients in a non-western context. In our context, medication beliefs are likely to have an independent role in effecting adherence to ASMs, particularly the concern that “ASMs disrupt life.” Treating physicians should cultivate good conscience about ASMs and evaluate the patient's medication beliefs early-on to identify those who might be at the risk of becoming nonadherent.

## 1. Introduction

The proper use of medications is critical towards effective self-management of chronic diseases, such as epilepsy [1]. For instance, intentional poor adherence to antiseizure medications (ASMs) [2] may exacerbate the impact of suboptimal care, such as a possible 21.0% increase in the risk of seizures [3]. This risk of poor adherence is more likely among elder adults owing to their possible fragile health, complex daily regimens, use of other medications, comorbidities, cognitive challenges, etc. [4].

Medication adherence is associated with numerous possible factors, of which include the social cognition theories of health behaviour. These self-regulatory theories take into account the patient's own evaluation of their medication [5]; which is fundamental to achieve good adherence beyond the perceptual and practical dimensions of the social, cultural, economic, and healthcare system contexts [6]. Broadly, the patient's perceptual framework towards medications can be categorized as the “need” and “concerns” [6]. This need-concern framework offers a convenient model for clinicians to underpin patients' attitudes and decisions about following prescribed treatment. In real-world as well, one may see that the patient's beliefs about medications differ for those who adhere than those who do not intentionally adhere [7]. Moreover, those with strong beliefs in the necessity of taking their medications are more adherent than those with higher levels of concerns [8].

However, medication beliefs are dynamic and may differ with the differing nature of the disease, type of medication, and sociocultural context of the patients. For example, in resource-poor populations, the patients may accommodate medicational side-effects more than those in the western populations, given the scarcity of therapeutic options for them [9]. Moreover, the entire attitude towards epilepsy differs from one to another population [10], and the general impression about ASMs is poor [11]. Thus, with such a vision, the primary objective of our study was to conduct a population-based survey in Tehran to determine beliefs about ASMs and their association with adherence to ASMs among elderly epilepsy patients. The secondary objective of our study was to enhance the psychometric properties and factor structure parameters of the Beliefs about Medications Questionnaire (BMQ). We expect that our work would help to provide additional ways to improve adherence and may, therefore, help to minimize early deaths and social burden among those with epilepsy [12].

## 2. Methods

We recruited eligible participants for this study from a population-based voluntary Tehran epilepsy register that has about 4800 epilepsy subjects of all kinds recruited from the general population. From this master list, we invited a sample of those aged at least 60 years for a free face-to-face consultation with the specialists as well as for the simultaneous collection of study data. Those who did not respond to our first invitation were contacted again for their participation. The eligible subjects were of any sex, any epilepsy and seizure type, under treatment with ASMs, resident of Tehran, and willing and able to participate independently. By using an expected correlation of 0.5 between medication beliefs and medication adherence, a type-I error of 0.01% and a type-II error of 0.1%, we required a total of 52 subjects [13].

All patients were carefully re-examined through a structured and reasonably detailed case-history examination. The criterion for epilepsy was a clear description of a recurrent and paroxysmal time-limited change in motor activity or behaviour. Consciousness was the major discriminator. The classification of seizures was done on the basis of information available from each subject to have a “best fit” seizure classification [14]. As per standard practice, we did not use electroencephalogram as a diagnostic modality. Besides, we did a cognitive assessment of all participants by using an Abbreviated Mental Test (AMT). Persian AMT has been found to have adequate psychometric parameters among older adults [15]. We did an assessment with AMT to determine the probable presence of morbid cognitive impairment among our participants.

Furthermore, we used the Persian version of Medication Adherence Rating Scale (MARS) and Beliefs about Medications Questionnaire (BMQ). The Persian language BMQ was used to measure the patient's beliefs about their need and concerns related to ASMs. This rating index is assessed by a five-item Likert-scale [16, 17] with a scale midpoint of 3.0. Higher the BMQ score, the stronger would be the belief about that particular item. The scores for each subscale of need and concern may range from five to 25. Ten items of BMQ questionnaire included considering all aspects of your epilepsy and its treatment with ASMs, please rate the following. Need subdomain includes the following: my health at present depends on my ASMs, my life would be impossible without my ASMs, without my ASMs, I would become very ill, my health in the future will depend on my ASMs, and my ASMs protect me from becoming worse. Concern subdomain includes the following: having to take ASMs worries me, I sometimes worry about the long-term effects of my ASMs, my ASMs are a mystery to me, my ASMs disrupt my life, and I sometimes worry about becoming too dependent on my ASMs.

MARS [18] is a 10-item multidimensional index that describes three binary dimensions: medication adherence behaviour (items 1–4), attitude toward taking medication (items 5–8), and negative side-effects and attitudes to psychotropic medication (items 9–10). Total MARS score may range between zero and ten, and a higher score indicates better adherence. Those with a MARS score of ≥6 were considered as adherent to their ASMs. MARS was used because it is considered as an improvement over other questionnaires on adherence and is reported to have better psychometric quality than other similar tools on adherence [18]. Since we used the previously established versions of MARS and BMQ, we did not conduct any face and content validity. However, the questionnaires we used had a minor addition of terms specific to epilepsy, for instance, the use of the term “ASMs” instead of “medicines.”

Both BMQ and MARS are well known within the medical literature, and their generic versions have been validated for the local Persian population and language [16, 19–22]. However, psychometric evaluation within the specific scope of epilepsy and affected elderly subjects remained unaddressed. Moreover, Cronbach alpha is often regarded as a sole uncritical index of validity; however, it is not so. There are other indices, such as item-test correlation that may ameliorate the problems with an indiscriminate use of Cronbach alpha as a sole index of reliability [23], for example, the item-test correlation, alpha coefficient by two subdomains of need and concern, and group-wise (≤scale midpoint≥) alpha coefficient.

All data were entered in MS-Excel and analyzed comprehensively. All data were described in descriptive terms, including the mean, proportion, 95% confidence interval (CI), and 5.0% statistical significance. First, we did a group comparison of means and proportions of all possible independent variables by our bivariate dependent variable (i.e., adherence) to identify those predictors that may have a significant difference between adherent and nonadherent. Then, to assess the relationship between adherence and BMQ items over the influence of other parameters, we did a hierarchical multiple linear regression. For this, independent predictor variables that had a significant group difference between adherent and nonadherent were classified into three different blocks. These blocks included the following: (a) sociodemographic characteristic block (Block-1), (b) treatment side-effect block (Block-2), and (c) BMQ block that included ten items of the BMQ scale (Block-3). Here, we observed any possible change in *R*^2^ between the three blocks since a significant increase in the adjusted *R*^2^ would mean that the predictor block improves the model more than by chance. We also did a forward step-wise linear regression in which the choice of independent variables is carried out by an automatic procedure by beginning with an empty model.

The psychometric properties of BMQ were determined by measuring the alpha coefficient for all and individual items, as well as for two subdomains of need and concern. We planned to extract those items with a low (<0.3) item-test correlation. We also measured the group-wise alpha coefficient separately, i.e., for responding ≤scale midpoint ≥to an item [16]. We did an exploratory factor analysis (EFA) to determine the possible number of latent factors that items, in turn, could be categorized into, by using eigenvalue ≥1.0 and factor loadings ≥0.30. For confirmatory factor analysis (CFA), we estimated the fit index, standardized size of the residual, and coefficient of determination. We evaluated sampling adequacy by using the Kaiser–Meyer–Olkin (KMO) test, and KMO measure more than 0.7 was considered as an appropriate measure for conducting a factor analysis. In addition, the data correlation matrix was tested with Bartlett's test of sphericity with a significance level of 5.0%. Lastly, we requested all potential participants to provide their informed verbal consent prior to participation. We obtained ethics permission from the institutional review board of the University of Social Welfare and Rehabilitation Sciences.

## 3. Results

**Figure 1:**
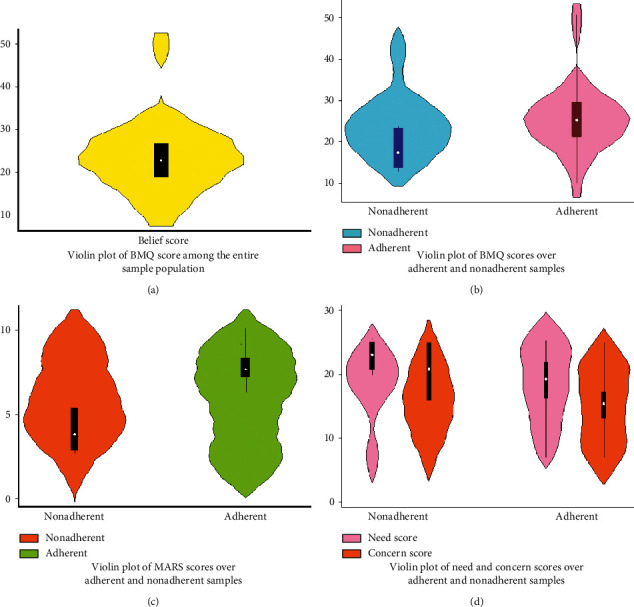
Violin plots summarizing various data points among elderly subjects with epilepsy.

**Figure 2:**
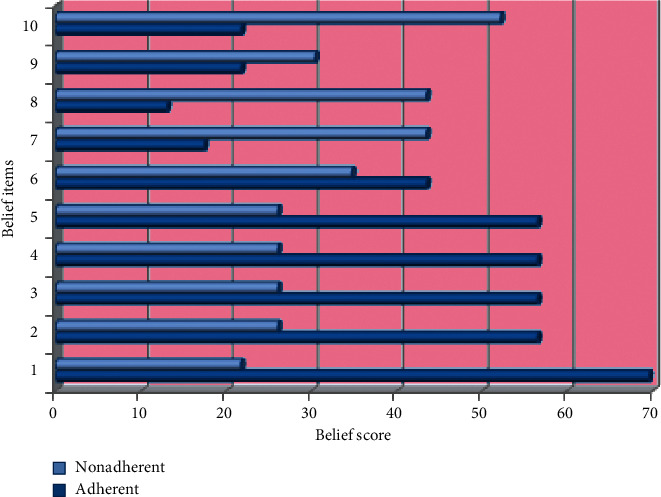
Distribution of belief items by adherent and nonadherent elderly subjects with epilepsy. The items include my health at present depends on my ASMs, my life would be impossible without my ASMs, without my ASMs, I would become very ill, my health in the future will depend on my ASMs, and my ASMs protect me from becoming worse, having to take ASMs worries me, I sometimes worry about the long-term effects of my ASMs, my ASMs are a mystery to me, my ASMs disrupt my life, and I sometimes worry about becoming too dependent on my ASMs.

**Figure 3:**
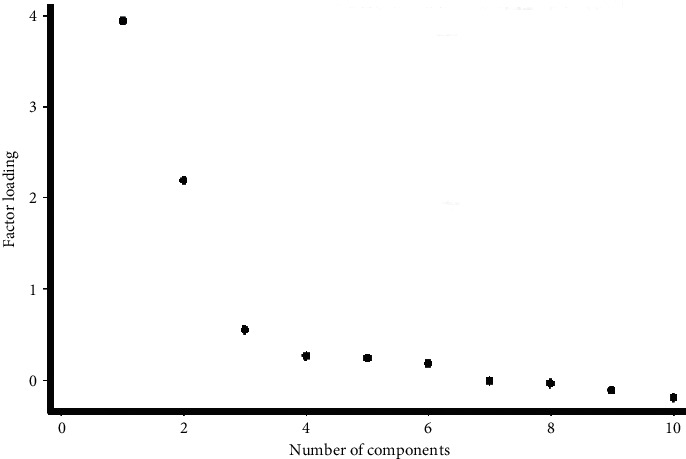
Screenplot showing the two-factor structure of the Beliefs about Medications Questionnaire.

We invited a total of 163 subjects for face-to-face consultation, of which 123 (mean age: 63.3, 95% CI 61.0–64.7, males: 73.9%) subjects successfully participated. The failure to participate was due to being in a debilitated state (*n* = 31, 77.5%), phone switched off, wrong or unreachable (*n* = 5, 12.5%), pollution (*n* = 3, 7.5%), and no one to accompany (*n* = 1, 2.5%). All results are summarized in Tables 1–3 and Figures 1–3 for convenience.

The majority of our participants (*n* = 96, 78.0%) were found to be adherent to their prescribed ASMs. The mean adherence score of the entire sample was 7.0 (95% CI 6.2–7.8). The mean MARS score was significantly different for those who are adherent and nonadherent (7.7, 95% CI 7.2–8.3 *vs.* 4.4, 95% CI 3.2–5.5, *p*=0.0001), with a score differential of 54.5% between adherent and nonadherent.

In the entire sample, the mean medication belief score was 23.4 (95% CI 19.8–27.0, range 10–50), while the mean score of need and concern subdomains was 20.0 (95% CI 18.0–22.0) and 16.5 (95% CI 14.3–18.7), respectively. The mean belief score was lower among nonadherent than adherent (16.2, 95% CI 11.4–20.9 *vs.* 25.4, 95% CI 21.4–29.4, diff = 44.2%), respectively, *p*=0.001. Nonadherent had a higher need (23.0, 95% CI 20.5–25.4 *vs.* 19.2, 95% CI 16.8–21.6, diff = 18.2%, *p*=0.05) and concern (20.8, 95% CI 16.5–25.0 *vs.* 15.3, 95% CI 12.9–17.7, diff = 30.4%, *p*=0.01) score than adherent, respectively. By sex, the mean score of need (19.9, 95% CI 17.4–22.4 *vs.* 20.3 95% CI 15.9–24.7, *p*=0.4) and concern (16.5, 95% CI 13.7–19.5 vs. 16.3, 95% CI 12-8-19.8, *p*=0.4) did not differ between males and females, respectively.

The observed proportion of need-related items (i.e., ≥scale midpoint) was “heath at present depend upon ASMs” (*n* = 112, 91.3%), “life impossible without ASMs” (*n* = 101, 82.6%), “to become ill without ASMs” (*n* = 101, 82.6%), “health in future depend on ASMs” (*n* = 101, 82.6%), and “ASMs protect from becoming worse” (*n* = 101, 82.6%), Table 1. Similarly, the observed proportion of concern-related items was “worry about becoming too dependent on ASMs in the long-term” (*n* = 96, 78.2%), “having to take ASMs worries” (*n* = 75, 60.8%), “ASMs disrupt life” (*n* = 69, 56.5%), and “sometimes worry about long-term effects of ASMs” (*n* = 62, 50.2%). Importantly, ASMs were not a mystery for the majority of our participants (*n* = 91, 73.9%). To summarize, an average 84.3% subjects reported necessity scores ≥scale midpoint while 63.9% subjects reported concern (i.e., ≥scale midpoint) with a need-concern differential of 20.4%.

Upon comparison of all possible independent social and clinical variables between adherent and nonadherent, only few predictor variables were found to differ, Tables 1 and 2. To assess the relationship between adherence and BMQ items over the influence of other factors (independent variables), we did a hierarchical multiple linear regression. For this, independent variables that had a significant group difference between adherent and nonadherent were classified into three different blocks: (a) sociodemographic characteristic block (Block-1), (b) treatment side-effect block (Block-2), and (c) BMQ block that included ten items of the BMQ scale (Block-3). Here, we noted that while the adjusted *R*^2^ for Block-1 was 33.8% (comorbidity, *ß* −1.9, *p*=0.03), it was 53.8% (dizziness *ß* −2.4, *p*=0.04) for Block-2 and 92.2% (belief item “life impossible without ASMs” *ß* −2.5, *p*=0.02 and “ASMs disrupt life” *ß* −1.8, *p*=0.04) for Block-3. Upon forward step-wise linear regression, we found that “ASMs disrupt my life” (*ß* −1.9, ES = −1.1, *p*=0.008) and comorbidity (*ß* 0.49, ES = 0.25, *p*=0.01) as the only factors found to be associated with adherence score.

The alpha coefficient of our BMQ questionnaire was 0.81, which was 0.82 and 0.69 for the subdomains of need and concern, respectively. The average item-test correlation of the entire BMQ was 0.62. Upon using our discriminant criteria of at least 0.3 item-test correlation for individual items, two items “ASMs protect me from becoming worse” and “ASMs are a mystery” were extracted from the model. The alpha coefficient of the revised BMQ questionnaire increased marginally to 0.83 (+2.5%), but increased considerably to 0.92 (+12.2%) and 0.83 (+20.3%) for need and concern subdomains, respectively. Furthermore, the item-test correlation of our entire BMQ increased by 9.7% from 0.62 to 0.68, while it increased by 13.9% from 0.79 to 0.90 and 18.2% from 0.66 to 0.78 for need and concern subdomains, respectively.

The alpha coefficient by ≤scale midpoint ≥to an item was found to be 0.89 and 0.82 for need and 0.81 and 0.83 for concern subdomains. Bartlett's test of sphericity was found to be significant (*x*^2^ = 756.5, *p*=0.001). Upon EFA, we found a two-factor structure of our BMQ with a cumulative variance of 87.2%, Figure 3. However, upon EFA of our revised BMQ, we noted a similar two-factor structure but with a higher cumulative variance of 99.5%. Upon CFA, we found a good model fit of our questionnaire with a CFI of 0.89, standardized residual of 0.1, and a coefficient of determination of 95.0%.

## 4. Discussion

Overall, we found that the majority of our participants were adherent to their ASMs. The belief score among nonadherent was lower by 44.2% than those who were adherent. Also, nonadherent had an 18.2% higher need and 30.4% higher concern than those who were adherent. Among our entire sample population, 84.3% subjects reported need score ≥scale midpoint while 63.9% subjects reported concern score ≥scale midpoint, meaning a positive need-concern differential of 20.4%. After controlling for social and clinical variables, adherence was associated with the beliefs that “ASMs disrupt life” and “life impossible without ASMs. Our BMQ questionnaire had an alpha coefficient of 0.83, while it was 0.92 and 0.83 for need and concern subdomains, respectively. We found a two-factor structure of BMQ with a 99.5% cumulative variance.

The methodology of our study was reasonably adequate. For example, our sample had community-dwelling residents nested in the general population pool. The focus of this study was on elder adults, given the increasing trend of aging in many developing countries, and inadequate prior assessment about the nature of their medication adherence behaviour and beliefs about ASMs [4]. Moreover, our study had both males and females, and patients of all epilepsy and seizure types. Besides the lack of adequate data about medication beliefs in epilepsy and elder subjects of non-western contexts [17, 24, 25], our study was necessary for many other reasons. For example, it is well known that despite numerous ASMs, many epilepsy patients never achieve (or maintain) their clinical remission [26]. So, a possible lack of achieving or maintaining a clinical remission in such patients may mean that adverse psychosocial sequelae would possibly be mediating their therapeutic outcomes [27].

We found that the majority of our subjects were adherent to their ASMs, while about a quarter of them were nonadherent. This result is in line with most other studies that have shown that about a quarter of epilepsy subjects may remain nonadherent for various reasons [1, 10]. In our study, excluding medication belief items, the adherence was found to be associated with comorbidity, which is a reasonably recognised factor in one's adherence to prescribed therapeutic regimes [4, 23]. The role of comorbidity in nonadherence is not necessarily intentional, because in the need of taking multiple tablets over a day, one also needs to maintain the time gap between the uptake of different medications to avoid interaction or adverse effects.

Nonadherent had a lower overall belief score and a higher mean concern score than those who were adherent. These results match with those of others that medication beliefs of adherent and nonadherent differ [7]. Upon step-wise logistic regression, “ASMs disrupt life” was the only belief item that was significantly associated with adherence. We believe this disruption in life could be due to a number of factors, such as adverse effects (Table 2), poor affordability [10], or patients' own faults as well [28]. Others have shown that some patients may have poor attitude towards their medications, and may view medications as the ones that prohibit their “life's pleasures” and “personal liberty” [29]. In our cultural context, such poor attitude of patients towards their medications may not be there. Iran has a hierarchical and traditional healthcare delivery model, in which the doctor-patient relation is mostly of unequal power dynamics [30] and patients are not necessarily the ones who ultimately decide whether or not they should adhere to the recommended regime [31].

Affordability is indeed a factor against adherence [10], but in Iran, nearly everyone is covered by a reimbursement system. Adverse effects could be the reason for poor adherence since several adverse effects were found to be significantly different between adherent and nonadherent, Table 2. Also, epilepsy is a pervasive illness and its long-term effects may complicate the already fragile bodily systems due to aging and other comorbid conditions and treatments. For instance, cognitive scores, although reasonably high, were different for adherent and nonadherent subjects. Such cognitive impairments may have occurred due to pervasive effects of epilepsy, due to ASMs or other treatments or due to natural processes (e.g., aging).

We estimated the psychometric properties of BMQ, particularly those that were previously unestimated. For example, by estimating group-wise alpha coefficient, we could see whether or not these coefficients differ between those responding ≤midpoint≥. Moreover, by using discriminant criteria such as the item-test correlation, we could purify the questionnaire by eliminating garbage items to have only those that are better representative of the underlying construct. This psychometric exercise was also necessary since BMQ had not been earlier adapted for the contexts specific to us, i.e., epilepsy and elderly subjects. Others have shown that item-level invariance may still be present even among validated questionnaires [32]. Moreover, estimating the psychometric properties was also essential to establish that the results we obtained were based on a questionnaire that was tested in the same population from which our other results were derived.

Lastly, our study was not designed for the future, but instead, we focused on addressing the immediate needs of the patients with epilepsy [33]. The relevance of the topic that our study addressed is reasonably broad as well since ASMs are also indicated for other disease conditions besides epilepsy. Our questionnaire includes common representations of need and concern regarding ASMs, and matches with relevant theoretical frameworks as well. One example is an appraisal theory of emotion, which asserts that our emotions (i.e., need and concerns) are determined by our appraisal of the stimulus (i.e., epilepsy).

However, many people with epilepsy could not participate, although we invited them twice. There are several tools for measuring medication adherence, but none of them is ideal [34]. Therefore, the results may vary depending upon the instruments used in the study for measuring adherence. We measured adherence alone, although there are additional concepts of compliance and concordance as well. Moreover, our results may also vary depending upon the level of power dynamics and concordance between treating physicians and their epilepsy patients. Similarly, the results may also vary with the frequency of need and concerns regarding ASMs [10]. For instance, in some cultural contexts, “*epilepsy is merely a problem of a few seconds, after which, one simply stands up and continues with what was left in-between”* (field data, D Bhalla 2014). Furthermore, we did not look at the role of many factors that may otherwise affect one's adherence such as religion training. We believe that religion training from the early age may help condition the psychopathological state of the affected patients [35]. Lastly, our study was based on patient-reported outcomes, but others have shown that the correlation between explicit and implicit measures are often quite small [36].

## 5. Conclusions

Ours is one of the very few studies that evaluated medication beliefs and their association with adherence to ASMs among elder epilepsy patients in a non-western context. In our context, we found that the need was higher than the concerns related to ASMs with a total need-concern differential of about 20.0%. Medication beliefs are likely to have an independent role in effecting adherence to ASMs. Treating physicians may benefit from evaluating their patient's medication beliefs early-on to identify those who might be at the risk of being nonadherent.

## Figures and Tables

**Table 1:** Sociodemographic characteristics of participants assessed for medication beliefs and adherence to antiseizure medications.

Parameter	Total (*N* = 123)	Adh (*N* = 96)	N-Adh (*N* = 27)	*p* value
*Comparison of group mean scores by using independent t-test:*				
AMT score	9.1 (CI 8.6–9.5)	9.3 (CI 8.9–9.7)	8.4 (CI 6.7–10.0)	ES = 0.4, *p* = 0.04
Distance^a^	1.2 (CI 0.6–1.8)	0.9 (CI 0.3–1.6)	1.9 (CI 0.4–4.3)	*p* = 0.09
Age	63.3 (CI 61.8–64.6)	63.1 (CI 61.6–64.5)	63.8 (CI 58.3–69.2)	*p* = 0.2
Family size	3.4 (CI 2.7–4.1)	3.5 (CI 2.7–4.4)	3.0 (CI 1.1–5.0)	*p* = 0.08

*Comparison of group proportions by using Z-test:*				
Married	78.3%	88.8%	40.0%	ES = 1.1, *p* = 0.001
Consang	26.1%	22.2%	40.0%	ES = 0.35, *p* = 0.03
Accept	82.6%	77.7%	100.0%	ES = 0.5, *p* = 0.001
Comorb	21.7%	5.5%	80.0%	ES = 2.2, *p* = 0.001
Male sex	73.9%	72.2%	80.0%	*p* = 0.20
Literate	95.6%	94.4%	100.0%	*p* = 0.10
Alone	13.0%	11.1%	20.0%	*p* = 0.10
FH	30.4%	33.3%	20.0%	*p* = 0.09
Supernat^*∗*^	52.2%	50.0%	60.0%	*p* = 0.20

*Note*. Accept: self-acceptance of epilepsy diagnosis; Adh: adherent; Alone: living alone; AMT: Abbreviated Mental Test; CI: 95% confidence interval; Comorb: patient-reported comorbidities; Consang: consanguinity; Distance: distance to nearest pharmacy in approximate KM; FH: family history of epilepsy; N-Adh: nonadherent; Supernat: a belief in the supernatural origin of epilepsy; *p* value: significance level of a group comparison of means or proportions.

**Table 2:** Clinical variables related to epilepsy among participants assessed for medication beliefs and adherence to antiseizure medications.

Parameter	Total (*N* = 123)	Adh (*N* = 96)	N-Adh (*N* = 27)	*p* value
*Comparison of epilepsy parameters by using Z-test:*				
Partial seizures	56.5%	50.0%	80.0%	ES = 0.52, *p* = 0.003
Mixed seizures	13.1%	16.6%	0.0%	ES = 0.42, *p* = 0.01
No group difference at 5.0% in generalized seizures, etiological types, and mean seizure frequency				

*Comparison of treatment parameters by using Z-test:*				
Valproic acid	43.5%	50.0%	20.0%	ES = 0.50, *p* = 0.003
Carbamazepine	43.5%	33.3%	80.0%	ES = 0.84, *p* = 0.001
Phenytoin	13.0%	16.6%	0.0%	ES = 0.42, *p* = 0.01
Others^a^	34.8%	34.8%	0.0%	ES = 0.70, *p* = 0.0002
No significant group difference at 5.0% in polytherapy and treatment with phenobarbital				

*Comparison of side-effect parameters by using Z-test:*				
Restlessness	13.0%	13.0%	0.0%	ES = 0.40, *p* = 0.02
Dizziness	17.4%	5.5%	60.0%	ES = 1.5, *p* = 0.001
Headache	26.1%	22.2%	40.0%	ES = 0.32, *p* = 0.03
Fatigue	43.5%	38.8%	60.0%	ES = 0.34, *p* = 0.02
No significant group difference at 5.0% in parameters of weight gain, nervousness, hyperactivity, hand tremors, poor attention, drowsiness, weight loss, appetite, upset stomach, double or blurred vision, and troubles with memory, sleep, mood, skin, and mouth and gums				

*Note*. Adh: adherent; ES: effect size; N-Adh: nonadherent; ^a^Others: primidone, clonazepam, lamotrigine, levetiracetam, and acetazolamide.

**Table 3:** Hierarchical regression related to epilepsy among participants assessed for medication beliefs and adherence to antiseizure medications.

Block-1	*ß*-Value	*p* value
AMT	0.42	0.21
Distance	−0.18	0.45
Marital status	−0.80	0.36
Consanguinity	−0.67	0.35
Acceptance of epilepsy	−0.42	0.65
Comorbidity	−1.9	0.03

Block-2	*ß*-Value	*p* value
AMT	0.37	0.21
Distance	−0.30	0.23
Marital status	−0.27	0.73
Consanguinity	−2.53	0.11
Acceptance of epilepsy	−0.59	0.55
Comorbidity	−0.88	0.34
Restlessness	−0.91	0.33
Dizziness	−2.41	0.04
Headache	1.65	0.15
Fatigue	−0.89	0.21

Block-3	*ß*-Value	*p* value
AMT	0.29	0.10
Distance	−1.01	0.02
Marital status	1.89	0.08
Consanguinity	0.89	0.12
Acceptance of epilepsy	−5.01	0.07
Comorbidity	−2.91	0.02
Restlessness	−0.99	0.70
Dizziness	2.41	0.04
Headache	1.83	0.08
Fatigue	2.33	0.07
My health at present depends on my ASMs	0.90	0.10
Life impossible without my ASMs	−2.46	0.02
Without my ASMs, I would become very ill	−0.16	0.26
My health in future depend on my ASMs	0.03	0.93
My ASMs protect me from becoming worse	0.20	0.14
I worry about becoming too dependent on my ASMs in the long-term	0.32	0.07
Having to take my ASMs worries me	0.25	0.10
My ASMs disrupt my life	−1.83	0.04
I sometimes worry about long-term effects of my ASMs	1.17	0.06
My ASMs are a mystery to me	0.82	0.07

*Note*. AMT: Abbreviated Mental Test; ASMs: antiseizure medications.

## Data Availability

All data are summarized in the manuscript.

## References

[1] Jones R. M., Butler J. A., Thomas V. A., Peveler R. C., Prevett M. (2006). Adherence to treatment in patients with epilepsy: associations with seizure control and illness beliefs. *Seizure*.

[2] Bhalla D., Lotfalinezhad E., Kapoor S. (2015). Anti-epileptic drugs: is terminology appropriate? A change might be needed. *Neurology Asia*.

[3] Al‐aqeel S., Al‐sabhan J. (2011). Strategies for improving adherence to antiepileptic drug treatment in patients with epilepsy. *Cochrane Database of Systematic Reviews*.

[4] Zeber J. E., Copeland L. A., Pugh M. J. V. (2010). Variation in antiepileptic drug adherence among older patients with new-onset epilepsy. *Annals of Pharmacotherapy*.

[5] Benedetti F., Carlino E., Pollo A. (2011). How placebos change the patient’s brain. *Neuropsychopharmacology*.

[6] Horne R., Cameron L. D., Leventhal H. (2003). Treatment perceptions and self regulation. *The Self-Regulation of Health and Illness Behaviour. 1*.

[7] Clifford S., Barber N., Horne R. (2008). Understanding different beliefs held by adherers, unintentional nonadherers, and intentional nonadherers: application of the Necessity-Concerns Framework. *Journal of Psychosomatic Research*.

[8] Foot H., La Caze A., Gujral G. (2015). The necessity-concerns framework predicts adherence to medication in multiple illness conditions: a meta-analysis. *Patient Education and Counseling*.

[9] Bhalla D., Aziz H., Bergen D. (2015). Undue regulatory control on phenobarbital-an important yet overlooked reason for the epilepsy treatment gap. *Epilepsia*.

[10] Hun C., Hok T., Bhalla D. (2014). Epilepsy: some controversies, some knowledge and some experience from Cambodia. *Neurology India*.

[11] Fainzang S. (2001). *Médicaments et Société. Le patient, le Médecin et L’ordonnance*.

[12] Dunbar-Jacob J., Erlen J. A., Schlenk E. A. (2000). Adherence in chronic disease. *Annual Review of Nursing Research*.

[13] Xu M. (2016). *Sample Size Formulas for Estimating Intraclass Correlation Coefficients in Reliability Studies with Binary Outcomes*.

[14] Commission on Classification and Terminology of the International League Against Epilepsy (1981). Proposal for revised clinical and electroencephalographic classification of epileptic seizures. *Epilepsia*.

[15] Foroughan M., Wahlund L.-O., Jafari Z., Rahgozar M., Farahani I. G., Rashedi V. (2017). Validity and reliability of abbreviated mental test score (AMTS) among older Iranian. *Psychogeriatrics*.

[16] Bakhtiyari F., Foroughan M., Fakhrzadeh H. (2014). Validation of the Persian version of abbreviated mental test (AMT) in elderly residents of Kahrizak charity foundation. *Iranian Journal of Diabetes and Metabolism*.

[17] Nakhutina L., Gonzalez J. S., Margolis S. A., Spada A., Grant A. (2011). Adherence to antiepileptic drugs and beliefs about medication among predominantly ethnic minority patients with epilepsy. *Epilepsy & Behavior*.

[18] Thompson K., Kulkarni J., Sergejew A. A. (2000). Reliability and validity of a new medication adherence rating scale (MARS) for the psychoses. *Schizophrenia Research*.

[19] Horne R., Weinman J., Hankins M. (1999). The beliefs about medicines questionnaire: the development and evaluation of a new method for assessing the cognitive representation of medication. *Psychology & Health*.

[20] Aflakseir A. (2012). Role of illness and medication perceptions on adherence to medication in a group of Iranian patients with type 2 diabetes. *Journal of Diabetes*.

[21] Arbabisarjou A., Zareban I., Dehghan M. (2016). Analytical assessment of belief about medicine among patients with hypertension: a case study on patients referred to medical centers. *Global Journal of Health Science*.

[22] Sanii Y., Torkamandi H., Gholami K. (2016). Role of pharmacist counseling in pharmacotherapy quality improvement. *Journal of Pharmacy Practice and Research*.

[23] Agbo A. A. (2010). Cronbach’s alpha: review of limitations and associated recommendations. *Journal of Psychology in Africa*.

[24] Shahin S. H., Daly E. B. (1999). Knowledge, attitudes and beliefs about psychotropic medication among Saudi hospitalized psychiatric patients. *International Journal of Nursing Studies*.

[25] Nguyen T.-M.-U., Caze A. L., Cottrell N. (2014). What are validated self-report adherence scales really measuring?: a systematic review. *British Journal of Clinical Pharmacology*.

[26] Hughes D. M., Bonnett L. J., Czanner G., Komárek A., Marson A. G., García-Fiñana M. (2018). Identification of patients who will not achieve seizure remission within 5 years on AEDs. *Neurology*.

[27] Jalava M., Sillanpää M., Camfield C., Camfield P. (1997). Social adjustment and competence 35 years after onset of childhood epilepsy: a prospective controlled study. *Epilepsia*.

[28] Nafradi L., Galimberti E., Nakamoto K. (2016). Intentional and unintentional medication non-adherence in hypertension: the role of health literacy, empowerment and medication beliefs. *Journal of Public Health Research*.

[29] Nations M., Firmo J. O. A., Lima-Costa M. (2011). Balking blood pressure “control” by older persons of Bambuí, Minas Gerais State, Brazil: an ethno-epidemiological inquiry. *Cadernos de Saúde Pública*.

[30] Barofsky I. (1978). Compliance, adherence and the therapeutic alliance: steps in the development of self-care. *Social Science & Medicine. Part A: Medical Psychology & Medical Sociology*.

[31] Donovan J. L., Blake D. R. (1992). Patient non-compliance: deviance or reasoned decision-making?. *Social Science & Medicine*.

[32] Schnettler B., Miranda-Zapata E., Lobos G. (2017). Cross-cultural measurement invariance in the satisfaction with food-related life scale in older adults from two developing countries. *Health Qual Life Outcomes*.

[33] Commission on Classification and Terminology of the International League Against Epilepsy (2002). Declaration of santiago on epilepsy in Latin America. *Epilepsia*.

[34] Farmer K. C. (1999). Methods for measuring and monitoring medication regimen adherence in clinical trials and clinical practice. *Clinical Therapeutics*.

[35] ASEM (2016). Approaches to reducing stigma. *Academies of Sciences E, and Medicine, Editor. Ending Discrimination against People with Mental and Substance Use Disorders: The Evidence for Stigma Change. 1*.

[36] Heine S. J., Lehman D. R. (1999). Culture, self-discrepancies, and self-satisfaction. *Personality and Social Psychology Bulletin*.

